# Solid Phase-Based Cross-Matching as Solution for Kidney Allograft Recipients Pretreated with Therapeutic Antibodies

**DOI:** 10.1155/2015/587158

**Published:** 2015-01-15

**Authors:** Gerald Schlaf, Susanne Apel, Anja Wahle, Wolfgang W. Altermann

**Affiliations:** Tissue Typing Laboratory, University Hospital Halle (Saale), 06112 Halle (Saale), Germany

## Abstract

In order to select recipients without donor-specific anti-HLA antibodies, the complement-dependent cytotoxicity crossmatch (CDC-CM) was established as the standard procedure about 40 years ago. However, the interpretability of this functional assay strongly depends on the vitality of isolated donors' lymphocytes. Since the application of therapeutic antibodies for the immunosuppressive regimen falsifies the outcome of the CDC-crossmatch as a result of these antibodies' complement-activating capacity in the recipients' sera, we looked for an alternative methodical approach. We here present 27 examples of AB0 blood group-incompatible living kidney allograft recipients who, due to their treatment with the humanized chimeric monoclonal anti-CD20 antibody Rituximab, did not present valid outcomes of CDC-based pretransplant cross-matching. Additionally, four cases of posttransplant cross-matching after living kidney allografting and consequent treatment with the therapeutic anti-CD25 antibody Basiliximab (Simulect) due to acute biopsy-proven rejection episodes are presented and compared regarding CDC- and ELISA-based crossmatch outcomes. In all cases, it became evident that the classical CDC-based crossmatch was completely unfeasible for the detection of donor-specific anti-HLA antibodies, whereas ELISA-based cross-matching not requiring vital cells was not artificially affected. We conclude that ELISA-based cross-matching is a valuable tool to methodically circumvent false positive CDC-based crossmatch results in the presence of therapeutically applied antibodies.

## 1. Introduction

It has been known for more than forty years that antibodies which are directed against HLA antigens of a given donor represent the dominating reason for hyperacute or acute rejections of renal allografts and allografts of other organs. These donor-specific anti-HLA antibodies (DSA) are thus regarded as a contraindication for grafting according to the transplantation guidelines of most countries and supranational societies (e.g., Eurotransplant) which are responsible for the supervision of the allocation of organs. In order to detect and exclude these harmful DSA, the so-called crossmatch (CM) procedure was developed in the late sixties of the last century. As standard technique to detect DSA, the complement-dependent cytotoxicity (CDC) assay was first established [1]. This test incubated donors' B- and T-cells with selected recipients' sera in the presence of rabbit complement. Consequently, this system is activated via the classical pathway of complement activation only by those antibodies which, in a first incubation step, have been bound to their target molecules on the donors' cells. The readout of this assay is performed by two-color fluorescence microscopy with definition of the reaction on the basis of a score system according to standard protocols of the National Institute of Health (USA). In this respect, the percentage of red cells lysed by the activated complement components and stained red by the intercalating agent ethidium bromide is indicated. Vital lymphocytes not recognized by a given recipient's antibodies exhibit a green staining pattern through the active uptake of acridine orange. Thus, as a functional assay, the CDC detects only antibodies which exert their detrimental function by their complement-fixing activity. However, this technique fails to identify donor-specific antibodies without complement-activating effector function although these may as well be detrimental for tissues/organs of a given donor. Another drawback of the CDC known for years is its low sensitivity leading to the general failure of detecting low concentrations of DSA. This drawback led to a modification of this assay using secondary anti-human IgG antibodies (termed AHG-enhanced CDC-CM) in order to enhance the complement activation and thus to increase the sensitivity of the CDC-CM [2, 3]. Flow cytometry-based crossmatch techniques which were alternatively implemented [4], however, have to be carefully interpreted due to other methodical difficulties. All over the years the outcome has artificially been influenced by the “irrelevant” binding of the recipients' antibodies to Fc-receptors highly expressed on B-lymphocytes, thus leading to many false positive results especially of B-cell cross-matching [5, 6]. An additional striking disadvantage which holds also true for CDC-based cross-matching is that both assays depend on the high vitality of donors' cells and do not lead to valid results if only cells of poor quality (vitality) are available. Due to this methodical drawback, novel methods which are characterized by complete independence of the cell quality were generated in the past. In this context, two assays have been developed using the design of enzyme-linked immunosorbent assays (ELISA) which are the AbCross HLA class I/II ELISA (Biotest, Dreieich, Germany) and the Antibody Monitoring System (AMS) HLA class I/II ELISA (GTI Diagnostics, Waukesha, USA). Both assays allow the detection of donor-specific antibodies by immobilizing detergent-extracted HLA molecules of chosen donors to precoated monoclonal capture antibodies. These are directed against monomorphic epitopes of HLA class I or II molecules, respectively. Due to the commercial availability of the AMS-ELISA as the first procedure, which exhibited complete independence of the donors' cell quality, this assay was first established in our tissue typing laboratory. Until its discontinuation by the manufacturer in 2013 for commercial reasons, it was used by us for many special diagnostic approaches which, for various reasons, did not result in valid CDC-CM outcomes [5, 7, 8]. Furthermore, this ELISA-based procedure was modified to be implementable for acellular tissue leading to the first crossmatch procedure exclusively using outer corneal rims as the only material available from a given donor's retain sample [9]. However, after its discontinuation by the manufacturer in 2013 solely for commercial reasons, this assay has been replaced by the alternative AbCross crossmatch ELISA (Biotest/Biorad, Dreieich, Germany) in a highly modified manner until now also leading to valid results. We here provide evidence that ELISA-based cross-matching represents a valuable tool to circumvent the interference of the therapeutically applied humanized chimeric antibodies Rituximab (anti-CD20) and Basiliximab (Simulect) (anti-CD25) generally not leading to valid outcomes if used prior to CDC-based cross-matching. Alternative solid phase-based cross-matching is shown as an adequate way to lead to valid detections of donor-specific antibodies in spite of Rituximab treatment generally used prior to AB0 blood group-incompatible living kidney donations and Basiliximab treatment in many transplant centers used to counter rejection episodes after kidney grafting.

## 2. Patients and Methods

### 2.1. Patients

All of the patients (*n* = 31) included in these comparative investigations were examined between February 2006 and May 2014 in the Tissue Typing Laboratory of the University Hospital Halle (GHATT) for DSA prior to AB0 blood group-incompatible kidney grafting (*n* = 27) or for possible DSA occurring during posttransplantation rejection episodes (*n* = 4). According to the German transplantation law and the guidelines of the transplant centers arranging the procedure of AB0 blood group-incompatible living kidney donations, at least two* de facto* (i.e., practical) and not virtual crossmatches are required prior to grafting with the last one always after treatment using the anti-CD20 mAb Rituximab. In this context, the AMS-ELISA has been used twenty-seven times to reliably exclude or detect DSA.

### 2.2. Detection of Donor-Specific Antibodies by Crossmatching

All of the patients were initially investigated for DSA by the standard CDC-CM procedure in detail described elsewhere [5, 8]. In spite of its drawbacks, the CDC-CM has been accepted for years and currently represents the standard procedure for the selection of donor-recipient combinations in order to exclude highly deleterious DSA. In accordance with most of the laboratories, this test is performed by us not only using the whole fraction of peripheral blood lymphocytes (PBL) but also using separated T- and B-cells. All approaches of cell isolation were performed using tetrameric antibody technique by crosslinking unwanted cells to red blood cells and subsequently eliminating them via density gradient centrifugation (System RosetteSep, Stemcell Technologies via CellSystems Biotechnology GmbH, St. Katharinen, Germany). Cells which have been recognized by DSA are consequently stained red by the DNA-intercalating agent ethidium bromide, whereas vital cells which have not been recognized are consequently stained green by the active uptake of acridine orange. The outcome, that is, the intensity of the complement reaction, is categorized through the percentaged indication of dead (red colored) cells using a score system of the National Institute of Health (Washington, USA) as shown: score 1: <10% (negative), score 2: 10–20% (doubtfully positive), score 4: 20–50% (weakly positive), score 6: 50–80% (positive), and score 8: 80–100% (strongly positive). It is noteworthy that the background of the CDC-CM caused by dead cells should not exceed 10% to get reliable results also of faint antibody-mediated reactions. Furthermore, antibodies detectable in this vitality assay must belong to the so-called cytotoxic, that is, complement-activating, isotypes IgM, IgG1, and IgG3 whereas other isotypes are not detectable.

**Figure 1:**
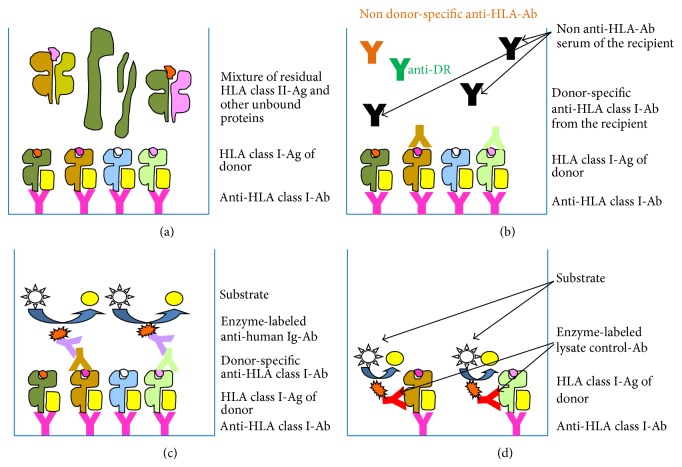
Flow diagram of the crossmatch-ELISA for the detection of donor-specific anti-HLA class I antibodies. (a) Binding of the donor's solubilized HLA class I molecules by monoclonal capture antibodies recognizing a monomorphic epitope on HLA class I molecules. (b) Binding of the donor-specific anti-HLA antibodies out of the recipient's serum to the HLA molecules of the donor. (c) Binding of enzyme-conjugated secondary anti-human IgG (anti-human IgG/M/A) antibodies to the bound recipient's donor-specific anti-HLA class I antibodies and subsequent color reaction. (d) Lysate control using an enzyme-conjugated monoclonal antibody directed against a second monomorphic epitope (AMS-ELISA) or the *β*2-microglobulin (AbCross) for detection in order to confirm the immobilization of a sufficient amount of HLA molecules by the capture antibody to generate a signal. The ELISA-variant for the detection of donor-specific anti-HLA class II antibodies is correspondingly designed.

As procedure of ELISA-based cross-matching, the Antibody Monitoring System (AMS) class I/II ELISA (GTI, Waukesha, USA, FDA-number BK 060038 from June 26, 2006) was first implemented by us until its discontinuation in 2013 when it had to be replaced by the alternative AbCross HLA class I/II ELISA (Biotest/BioRad, Dreieich, Germany). In order to lead to a higher sensitivity and to considerably faster results, the laborious procedure presented in the manual of the manufacturer was completely modified by us resulting in a workflow which was very similar to that of the former AMS ELISA. Both assays, however, reliably allowed the direct detection of DSA by immobilizing extracted HLA molecules from donor cells/tissues onto which, in a consecutive step, only donor-specific but not anti-HLA antibodies in general out of a given recipient's serum bind. The principle of work is demonstrated in the flow scheme (Figure 1). Detergent lysate of the donor's leukocytes/tissue including HLA class I and class II molecules has first to be filled into the wells of ELISA strips (GTI) or Terasaki-Microtest plates (BioRad), respectively, precoated with monoclonal antibodies (Figure 1(a)). These are directed against monomorphic epitopes available on all HLA class I or class II molecules, respectively. After this first incubation and subsequent washing steps, the recipients' sera are pipetted onto the immobilized HLA molecules and, in case of recognizing them, serve as detection antibodies in this sandwich assay (Figure 1(b)). Upon rewashing, the samples are incubated with enzyme-conjugated secondary anti-human IgG (alternatively anti-human IgG/M/A) antibodies which induce the final substrate reaction (Figure 1(c)). Of high relevance are the so-called lysate controls (positive controls) of both ELISA-based crossmatch assays, which consist of a second enzyme-labeled monoclonal antibody, thus providing evidence that a sufficient quantity of the donor's HLA molecules has been immobilized to reach a significant signal (Figure 1(d)). To be classified as positive, the value of a given recipient's serum sample has to exceed twofold the value of the negative control serum. ELISA-based cross-matching was implemented in our tissue typing laboratory more than eight years ago and has become a reliable diagnostic crossmatch tool by reinvestigating nearly all samples which have been characterized by doubtful or invalid outcomes of the conventional CDC-CM.

### 2.3. Procedures Used for the Detection/Specification of Anti-HLA Antibodies (Antibody Monitoring) in order to Verify the Results of CDC- and ELISA-Based Cross-Matching

The recipients' sera were generally screened for anti-HLA class I antibodies using the QuikScreen ELISA (BioRad) and for anti-HLA class II antibodies using the B-Screen ELISA (BioRad). Serum samples positive in this first screening step were afterwards investigated for antibody specification using the DynaChip HLA antibody analysis technique (Invitrogen/Dynal, Bromborough, UK) until 2011 when this system was discontinued by the manufacturer. This miniaturized chip-based technique was the only completely automated system available for the detection and specification of anti-HLA antibodies. In its second generation design 106 positions of each microchip were covered with HLA class I and 48 positions with HLA class II molecules of different single donors, respectively. Thus, apart from a number of HLA class II DQ-antigens immobilized on some positions, this assay did not provide a resolution at the single antigen level. However, the combination of the single donors' immobilized HLA class I or II antigens, respectively, allowed the identification of the patients' antibody specificities in most cases (70–80%) especially if the immunization level/PRA-level (see below) was not too high. The system, however, was discontinued by the manufacturer in 2011 for commercial reasons leading to the implementation of the Luminex technique in our laboratory. This technique currently represents the dominating tool for anti-HLA antibody specification. Its technical aspects and drawbacks for antibody specification have in detail been reviewed and discussed elsewhere [8, 10, 11]. Depending on its availability during the last years, first the DynaChip and afterwards the Luminex technique were used for anti-HLA antibody specification in order to perform the approach of virtual cross-matching, that is, the identification of anti-HLA antibody specificities directed against phenotypes of a given donor. The general degree of anti-HLA presensitization termed “panel reactive antibodies” (% PRA) was also defined using both techniques at the single donor (single ID) level. Originally, this percentage PRA-value had been defined by cell tray analysis as CDC-based reactivity against either a cell panel of PBL or a cell panel from various chronic lymphatic leukemia (CLL) patients. As a matter of course, any panel has to comprise all HLA phenotypes of a given recipient's population and to represent the phenotypes' frequencies of this patient's population. The statistical PRA-value which is quarterly determined for all patients of the kidney waiting list indicates the likelihood of an individual positive* de facto* crossmatch and is not to be equated with DSA. Thus, a high value [%] easily allows the identification of those patients who have to be monitored and crossmatched very carefully as a consequence of a high anti-HLA preimmunization status.

## 3. Results and Discussion

### 3.1. False Positive CDC-Based Crossmatch Results in Patients Pretreated with the Therapeutical Antibodies Rituximab and/or Basiliximab

During the last decade, humanized monoclonal antibodies have increasingly been used for preconditioning AB0 blood group-incompatible recipients of living kidney donations (anti-CD20/Rituximab) or for the therapy of acute rejection episodes (anti-CD25/Basiliximab). Rituximab which was originally used to administer a therapy against B-cell non-Hodgkin's lymphoma [12] was afterwards implemented to defend recipients against humoral rejections in general but especially to eliminate naturally occurring antibodies highly deleterious in approaches of AB0 blood group-incompatible kidney donations [13–15]. All of the four kidney transplant centers which are in contract with our tissue typing laboratory have implemented the procedure of AB0 blood group-incompatible kidney donations by preconditioning the recipients with the therapeutical anti-CD20 mAb Rituximab. One transplant center, however, stopped this procedure in 2011 due to severe side effects of the Rituximab application leading to three centers not frequently employing this procedure. Hitherto a total of 27 blood group-incompatible donations under Rituximab application have been performed (Table 1). According to the German transplantation law and the guidelines of the different kidney transplantation centers, at least two* de facto*/practical crossmatches are required prior to the living kidney donation. In this context, it became evident for us right from the beginning of AB0 blood group-incompatible living kidney grafting in 2006 that CDC-based cross-matching may be highly influenced by Rituximab. As is visible in Table 1, all of the cases shown were characterized by a clearly positive outcome with B-cells highly or maximally attacked, that is, strongly positive as shown by scores between 6 and 8. These scores were observable although the great majority of the patients (24 out of 27, i.e., 89%) did generally not exhibit anti-HLA antibodies and were thus characterized by a PRA value of 0%. Only three of the patients (11%) were characterized by anti-HLA antibodies as shown by relatively low PRA-levels between 4% and 18%. These three patients, however, did not exhibit donor-specific antibodies as was demonstrable by virtual cross-matching, thus confirming the negative outcome on donor-specific anti-HLA antibodies determined by ELISA-based cross-matching (Table 1). Hence, the data provide clear evidence that the positive reactions of the CDC-based crossmatch outcomes were not the result of donor-specific anti-HLA alloantibodies but were due to the therapeutic anti-CD20 monoclonal antibody (moAb) Rituximab as part of the recipients' sera. The results of Table 1 become clear because the therapeutical monoclonal Rituximab belongs to the IgG1 isotype which is capable of inducing the activation of the complement system responsible for positive reactions in CDC-based assays. Thus, the corresponding positive results with scores between 6 and 8 represented the recognition by the therapeutical anti-CD20 moAb leading to the complement-mediated lyses of isolated B-cells (scores 6 to 8) or the lysed fraction of B-cells out of total PBL with scores between 2 and 2/4 depending on individually different percentages of B-cells (5–15% of PBL).

Interestingly, also the fraction of isolated T-cells in most cases appeared slightly positive (scores between 1/2 and 2) due to an apparent drawback of the RosetteSep cell separation system by Stemcell Technologies. This puzzling factor of residual B-cells in the fraction of isolated T-cells was presented and discussed a few years ago [16]. Most probably, it is due to an insufficient amount of bispecific anti-CD19 antibodies (among other monoclonal antibodies) crosslinking unwanted B-cells to human red blood cells by forming tetrameric antibody complexes. This drawback has apparently not been stopped by the manufacturer until now. Thus, donor-specific anti-HLA class II antibodies may generally lead to a pseudopositivity of T-cells hampering the interpretability of DSA detectable by CDC-CM between a given recipient and her/his donor. Both ELISA-based crossmatch systems, the AMS and the AbCross, however, have resulted in reliable and plausible results in best accordance with the virtual antibody analyses and consequently represent an adequate procedure to circumvent artefacts through the use of Rituximab.

As part of these investigations, the other therapeutical moAb Basiliximab (Simulect) which is directed against the alpha-chain of the interleukin 2 receptor (CD25) [17–20] influenced the results of CDC-based cross-matching more or less evenly in all three cell populations under investigation. As is visible in the lower part of Table 1 (Basiliximab posttransplant group), it was more complex to identify an artificial, that is, Basiliximab-mediated, effect on the outcome of the CDC since three of the four patients were characterized by anti-HLA antibodies and all of them were retrospectively investigated for anti-HLA antibodies due to bioptically and clinically proven posttransplant rejection episodes. However, including the results of virtual cross-matching, the ELISA-based crossmatch results were plausible in contrast to the CDC-based data. Although the PRA-level of patient 2 was very high (i.e., 86%), no donor-specific antibodies were demonstrable both by ELISA-based and by virtual cross-matching leading to the conclusion that indeed no donor specific anti-HLA antibodies exist in spite of the high degree of immunization. Most probably, HLA molecules were not the target of the bioptically proven rejection.

In the same context, patient 1 was important as she/he did generally not exhibit any anti-HLA antibodies (PRA = 0%) in best accordance with negative outcomes of ELISA-based cross-matching. These negative outcomes clearly contrasted with the positive CDC-based crossmatch results of isolated T-cells, B-cells, and PBL strongly suggesting these data as artificially positive. The CDC-based crossmatch outcomes of patients 3 and 4 were again not plausible in contrast to the ELISA-based ones. In best accordance with the results of virtual cross-matching, the ELISAs clearly exhibited donor-specific anti-HLA class II antibodies. CDC-based cross-matching, however, led to implausible positive results including PBL and T-cells as target cells, thus not indicating donor-specific antibodies solely directed against HLA-class II molecules. Concludingly, the positive outcomes of CDC-based cross-matching of patients 3 and 4 were rather the result of the complement activation induced by the therapeutic Basiliximab antibody (isotype IgG1).

In spite of the limited number of only four cases presented in the context of posttransplant cross-matching under the immunosuppressive regimen of Basiliximab, the cases show that ELISA-based cross-matching represents a valid procedure to circumvent artificial outcomes induced by this therapeutical moAb which, for all these patients, has been applied to avoid the loss of kidney allografts due to clinically apparent rejection episodes. As a matter of course, fresh donor cells have to be available for any CDC-based cross-matching procedure. Thus, the comparative posttransplant investigations which comprised both crossmatch procedures were limited to living kidney donations for which a given donor's material was again available for successive investigations after the kidney donation. Specifically due to this fact, only four cases were suitable for their observance in this group of patients.

Generally, it is noteworthy that the procedure of ELISA-based cross-matching does not require vital cells of the donor but advantageously uses detergent lysates of the respective donors (Figure 1(a)). In this respect, it is not a problem to adequately store deep-frozen detergent lysate of the respective donors as retain sample for several years. Consequently, ELISA-based cross-matching first provides an adequate diagnostic tool to perform* de facto*/practical instead of virtual cross-matching through the use of a given donor's retain sample consisting of nonvital, that is, in the long run storable material [5, 7–9]. Hence, this procedure additionally allows retrospective* de facto*/practical cross-matching after a cadaver organ donation, that is, in a situation which is regularly limited to virtual cross-matching since vital donors' cells are not available after transplantation. To our best knowledge, the only approach to perform a retrospective* de facto*/practical crossmatch was published about eighteen years ago in the context of corneal grafting using a flow cytometry-based procedure. As donor's postmortem material cultured retinal pigment epithelial cells taken from explanted eyes of the donors were used [21]. For this purpose, the isolated and deep-frozen cells had to be cultured and stimulated with recombinant IFN-*γ* to upregulate the expression of HLA-molecules for the subsequent cross-matching. However, this historical approach must be regarded as time consuming, expensive, and technically very challenging in contrast to the ELISA-based procedure described here in the context of therapeutically applied antibodies which is indeed easily implementable into the routine task of any tissue typing laboratory. As this procedure does not require vital lymphocytes or other isolated vital cells in general, it even allows the use of any residual and acellular tissue such as blood vessels or corneal material [7–9].

Interestingly, the aspect of medical treatment falsifying the outcome of CDC-based cross-matching has hitherto been described only a few times. This holds true for patients suffering from various forms of leukemia thus destined for a transfer of hematopoietic stem cells. Regularly, these patients do not have to fulfill the criterion of a negative crossmatch prior to the transfer since the recipient and her/his chosen donor have to be identical at the high resolution (four digits) level of HLA-typing. However, cases exist where the transfer is performed between two persons not completely HLA-identical in all genotypes or only identical for one HLA-haplotype if the donation is, for example, arranged between parents and their children (haploidentical donation). These configurations may lead to the requirement to exclude donor-specific anti-HLA antibodies. In this context, false-positive CDC-based crossmatch outcomes were reported to be the result of an unspecific cell death caused by the cytostatic agent 6-mercaptopurine which was used to administer a therapy against leukemia [22]. The alternative application of ELISA-based cross-matching, however, led to the clear exclusion of DSA in best accordance with the results of virtual crossmatch analyses.

The diagnostic interference of the therapeutic humanized chimeric moAbs Rituximab and Basiliximab leading to false-positive results, mainly of the CDC-CM, but also of the flow cytometry-based crossmatch results, was first described about nine years ago [23]. In contrast to these standard assays' falsified outcomes, the Transplant Monitoring System (TMS) as precursor system of the Micro-AMS ELISA afterwards used by our laboratory was the only system which was not artificially influenced by those therapeutic moAbs. However, due to the old name “TMS-ELISA,” we were not aware of the investigations of Book and coworkers for years. As a matter of fact, we independently developed the idea to implement ELISA-based cross-matching in order to exclude donor-specific antibodies for AB0 blood group-incompatible living kidney donations using the AMS-ELISA (GTI, Waukesha, USA) already in the year 2006. Right from the beginning of this procedure in the kidney transplant centers of our area, we were aware of the problem that CDC-based cross-matching of recipients preconditioned with Rituximab would never result in the detection of HLA-specific alloantibodies but exhibited only this moAb's B-cell depleting activity.

In conclusion, the results of these investigations indicate the urgent need to implement ELISA-based cross-matching for the reliable exclusion of DSA (i) in the increasing field of AB0 blood group-incompatible living kidney donations and (ii) for any situation of cross-matching under the regimen of therapeutic antibodies not allowing the application of a cellular vitality assay such as CDC-based cross-matching.

## Figures and Tables

**Table 1:** Comparison of the outcome of CDC-based cross-matching with ELISA-based cross-matching (AMS- or AbCross-ELISA, resp.) as shown for twenty-seven patients treated with anti-CD20 mAb Rituximab and four patients treated with anti-CD25 mAb Basiliximab (Simulect).

Patient's number	CDC-CM (NIH-score)			ELISA-CM		Antibody detection/specification (PRA max.)
	PBL	T-cell	B-cell	Class I	Class II	
Rituximab (anti-CD20) [AB0-incompatible living kidney donations] pretransplant						
(1)	2	1/2	6/8	neg.	neg.	PRA = 0%
(2)	2	1	6	neg.	neg.	PRA = 0%
(3)	2/4	1/2	8	neg.	neg.	PRA = 0%
(4)	2	1/2	6/8	neg.	neg.	PRA = 0%
(5)	2/4	2	8	neg.	neg.	PRA = 0%
(6)	2	1/2	8	neg.	neg.	PRA = 0%
(7)	2/4	1/2	8	neg.	neg.	PRA = 0%
(8)	2	1/2	6/8	neg.	neg.	PRA = 0%
(9)	2	1/2	8	neg.	neg.	PRA = 0%
(10)	2/4	2	8	neg.	neg.	PRA = 18%^#^
(11)	2	1/2	6/8	neg.	neg.	PRA = 0%
(12)	2	1/2	6	neg.	neg.	PRA = 0%
(13)	2/4	2	8	neg.	neg.	PRA = 0%
(14)	2	1/2	6/8	neg.	neg.	PRA = 0%
(15)	2	1/2	8	neg.	neg.	PRA = 4%^#^
(16)	2	1	8	neg.	neg.	PRA = 0%
(17)	2	1/2	6/8	neg.	neg.	PRA = 0%
(18)	2	1	8	neg.	neg.	PRA = 0%
(19)	2/4	1/2	8	neg.	neg.	PRA = 12%^#^
(20)	2/4	2	6/8	neg.	neg.	PRA = 0%
(21)	2	1/2	8	neg.	neg.	PRA = 0%
(22)	2/4	2	8	neg.	neg.	PRA = 0%
(23)	2/4	1/2	6/8	neg.	neg.	PRA = 0%
(24)	2	1	6/8	neg.	neg.	PRA = 0%
(25)	2/4	2	8	neg.	neg.	PRA = 0%
(26)	2	1/2	8	neg.	neg.	PRA = 0%
(27)	2	1/2	6/8	neg.	neg.	PRA = 0%

Basiliximab (anti-CD25) posttransplant diagnostics of living kidney donations						
(1)	2/4	2/4	4	neg.	neg.	PRA = 0%
(2)	2/4	2/4	4/6	neg.	neg.	PRA = 86%^#^
(3)	4	4	6	neg.	pos.	PRA = 12%^&^
(4)	6	4	6/8	neg.	pos.	PRA = 20%^&^

The outcomes of CDC-based and ELISA-based cross-matching are compared by showing the respective NIH-scores (introduced in Section 2.2) and the corresponding ELISA-based results (introduced in Section 2.2). Additionally, the maximal level of panel reactive antibodies [PRA max.] (introduced in Section 2.3) of each patient is indicated exhibiting the highest historical individual level of immunization against HLA-antigens of the quarterly antibody screening runs. ^#^No donor-specific antibodies were identifiable by virtual cross-matching using the Luminex- or DynaChip specifications in spite of the general preimmunization (positive PRA-value); ^&^donor-specific **anti-HLA class II** antibodies were identifiable by virtual cross-matching using Luminex- or DynaChip specifications.
